# Sex-specific foraging behavior in response to fishing activities in a threatened seabird

**DOI:** 10.1002/ece3.1492

**Published:** 2015-05-22

**Authors:** Manuel García-Tarrasón, Juan Bécares, Santiago Bateman, José Manuel Arcos, Lluís Jover, Carolina Sanpera

**Affiliations:** 1 Departament de Biologia Animal, Facultat de Biologia, Universitat de BarcelonaAv. Diagonal 643, 08028, Barcelona, Spain; 2 Sociedad Española de Ornitología (SEO/BirdLife)C/Murcia 2-8 local 13, 08026, Barcelona, Spain; 3 Departament de Salut Pública, Facultat de Medicina, Universitat de BarcelonaC/Casanova 143 5a planta, 08036, Barcelona, Spain; 4 Institut de Recerca de la Biodiversitat (IRBio), Universitat de BarcelonaAv Diagonal 643, 08028, Barcelona, Spain

**Keywords:** Audouin's gull, ban on discards, common fisheries policy reform, foraging behavior, habitat use, seabirds, sexual segregation

## Abstract

Some seabird species have learnt to efficiently exploit fishing discards from trawling activities. However, a discard ban has been proposed as necessary in Europe to ensure the sustainability of the seas. It is of crucial importance for the management and conservation purposes to study the potential consequences of a discard ban on the foraging ecology of threatened seabirds. We assessed the influence of fishing activities on the feeding habits of 22 male and 15 female Audouin's gulls (*Larus audouinii*) from the Ebro Delta (Mediterranean Sea) during the breeding period using GPS loggers together with Stable Isotope Analysis (SIA), which provided new insights into their foraging behavior and trophic ecology, respectively. GPS data revealed different sex-specific foraging patterns between workdays and weekends. Females were highly consistent in that they foraged at sea throughout the week even though discarding stops at weekends. In contrast, males switched from foraging at sea during the week (when discards are produced) to an increased use of rice field habitats at weekends (when fishermen do not work). This sex-specific foraging behavior could be related to specific nutritional requirements associated with previous egg production, an energetically demanding period for females. However, on a broader time scale integrated by the SIA, both sexes showed a high degree of individual specialization in their trophic ecology. The need to obtain detailed information on the dependence and response of seabirds to fishing activities is crucial in conservation sciences. In this regard, sex-specific foraging behavior in relation to fisheries has been overlooked, despite the ecological and conservation implications. For instance, this situation may lead to sex differentiation in bycatch mortality in longlines when trawlers do not operate. Moreover, any new fisheries policy will need to be implemented gradually to facilitate the adaptation of a specialized species to a discard ban scenario.

## Introduction

Industrialization of commercial fisheries over the last century has strongly impacted the marine environment (Pauly et al. 1998; Bicknell et al. 2013; Coll et al. 2013). Scavenger seabirds have often taken profit of this situation, learning to exploit new food resources provided by these human activities (as fishing discards) and modifying their foraging behavior to associate with fishing vessels (Granadeiro et al. 2011, 2014; Torres et al. 2011; Votier et al. 2013). Consequently, this situation has led to changes in seabird movement patterns (Bartumeus et al. 2010; Votier et al. 2010), wintering strategies (Hüppop and Wurm 2000), and breeding success (Oro et al. 1996a) with evident demographic consequences (see references in Oro et al. 2013). This is the case of many gulls and skuas, which have learnt to efficiently take advantage of the increasing amounts of fishing discards as an alternative to their declining natural prey (Arcos 2001; Furness 2003). However, this situation is unsustainable in the long run, due to fisheries overexploitation, and increasing efforts are being taken by fisheries managers to reduce this waste of fish (Penas 2007).

One of the major changes that will affect European marine ecosystems in a near future is the regulatory reform of the Common Fisheries Policy adopted in 2013 by the European Union (Penas 2007; http://ec.europa.eu/fisheries/reform/). One of its proposals is a ban on discards to ensure that fisheries are economically and environmentally sustainable (Bicknell et al. 2013). As many seabirds have learnt to exploit discards to such an extent that they now constitute the bulk of their diet, a discard ban may result in a food shortage and therefore a nutritional shortfall (Bicknell et al. 2013). However, some studies have shown that when discards decrease, seabirds respond by switching their diet to alternative prey (Oro et al. 1996a) or moving to novel habitats (Camphuysen et al. 2010). Therefore, it is of crucial importance for the management of endangered seabird species and the whole marine ecosystem to anticipate environmental consequences by generating information upon which to design and implement effective conservation measures (Orians and Soulé 2001).

A representative example of a seabird that has taken advantage of fishing discards is the threatened Audouin's gull (*Larus audouinii*), endemic to the Mediterranean. Although traditionally considered to be a nocturnal specialist forager on small pelagic fish and one of the most endangered seabirds in the world (Burger and Gochfeld 1996), its population has increased significantly as a new colony was established in the Ebro Delta (northeast of the Iberian Peninsula) in 1981 (Genovart et al. 2008). This population, which nowadays holds more than 50% of the world total of 22,000 breeding pairs, grew as a result of an efficient utilization of trawling discards, together with effective protection measures of the breeding grounds (Oro and Ruxton 2001; BirdLife International 2012). Fishing discards currently account for more than 75% of the energy required by this gull and represent the bulk of its marine diet (Arcos 2001; Oro et al. 2013). Alternative foraging opportunities, which become more important when trawlers do not operate, include the capture of small pelagic fish, both directly and from purse-seine vessels (Arcos and Oro 2002), and the use of rice fields (Navarro et al. 2010). Its main prey in the rice fields is the American crayfish (*Procambarus clarkii*), an abundant invasive species (Ruiz et al. 1996), although depleted in terms of nutritional content compared to fish (Hunner 1988; Massias and Becker 1990). For its conservation, the foraging behavior and habitat use of the Audouin's gull needs to be studied at both the population and individual level. In the Ebro Delta, fishing vessels (both trawlers and purse-seiners) operate from Monday through Friday with a defined daily regime of activity. Trawlers are restricted to operate during daylight hours, while purse-seiners operate at night and dawn. For the above reasons, weekend periods (when fishing activity and discard production is negligible) serve as a suitable time frame to study possible behavioral and dietary changes in a discard ban scenario (Bartumeus et al. 2010).

The study of seabird foraging behavior has always been hampered by the problem of assessing at-sea distribution, individuals being particularly difficult to track (Votier et al. 2010). In breeding Audouin's gulls, previous studies have addressed this issue at the population level (Arcos and Oro 1996; Cama et al. 2013), highlighting the species’ strong dependence on fishing activities, which shapes its at-sea distribution. However, the knowledge gap is especially obvious at an individual level. Other studies on the same species (Mañosa et al. 2004; Christel et al. 2012) have used tracking devices (radio tracking and PTTs loggers, respectively) with several positioning restrictions and errors (Burger and Shaffer 2008). As the Ebro Delta holds a great diversity of habitats in a relatively small area, slight positioning errors could result in considerable inaccuracies in determining foraging behavior. Recent improvements in GPS logging devices have revolutionized the study of free-living animal movement (Ropert-Coudert and Wilson 2005). In addition, diet tracers such as Stable Isotope Analysis (SIA), especially of carbon and nitrogen isotopes, are nowadays widely used to assess trophic ecology in a precise period, depending on the analyzed tissue (Kelly 2000). For example, it is well known that marine habitats present enriched carbon signatures compared to freshwater (Cotin et al. 2011; García-Tarrasón et al. 2013). The integration of positional information with SIA is becoming an increasingly used tool in disentangling the foraging behavior of seabirds and designing new conservation measures.

In this study, we used the information provided by GPS loggers and plasma SIA of the Audouin's gull to: (1) describe the habitat use and foraging behavior of each sex according to fish discard availability (workdays *vs*. weekends); (2) understand the degree of specialization in foraging behavior and habitat use among individual gulls; and (3) infer the relationship between foraging strategy and body condition, if any. Due to the great dependence of the Audouin's gull on commercial fisheries and considering the potential consequences of the reform of the EU fisheries policy, we hypothesize a greater foraging effort in absence of trawling activity, together with an increasingly detrimental effect of rice field resources (usually with a low energy content).

## Methods

### Tagging and sampling

The work was conducted at the Ebro Delta colony (NE Iberian Peninsula: 40°33′ N, 00°39′ E) during the 2011 breeding season. The Audouin's gull nesting colony is situated at Punta de la Banya, a protected sandy peninsula subject to salt works. From May 8th to 10th, 60 adult Audouin's gulls with three-egg clutches at the beginning of the incubation period were caught at the nest using cage traps. The birds were tagged with a GPS logger (CatTraq™) fixed with harnesses by a trained technician from the Spanish Ministry of Environment (MAGRAMA). The weight of the devices once placed on the bird was about 25 g, <5% of the specimen weight (Cochran 1980). To reduce any negative effect on the breeding performance of the birds, only one member of the pair was tagged (Mañosa et al. 2004). The harnesses were devised to last only a few months, so any unretrieved birds would lose the device after this time. The loggers were programmed to register positions every 5 min. A total of 37 birds (22 males and 15 females) were recaptured at the nest from May 18th to 26th, and the loggers were retrieved (ranging from 9 to 18 days between captures). Among the other 23 nests included in the study, 5 were considered abandoned at recapture time and in 18, we decided not to recapture to prevent major nuisances due to the mistrust these animals showed against the traps.

Adults were weighed both at capture and recapture to the nearest 10 g with a 1000 g Pesola spring balance. The skull length was measured at recapture to the nearest 0.01 mm using a digital calliper. Blood samples (0.5 mL) were collected from the brachial vein at both events and placed in heparinized vials for isotopic analysis. Recent studies recommend the exclusive use of heparin as a blood anticoagulant for field studies where prompt centrifugation is not possible, because it is the only anticoagulant with no measured effect on the blood plasma isotopic signature (Lemons et al. 2011). At the first capture, 0.2 mL was preserved in a neutral vial for molecular identification of sex. Sex was determined using polymerase chain reaction (PCR) amplification of the CHD genes (Griffiths et al. 1998).

### Positional information

**Figure 1:**
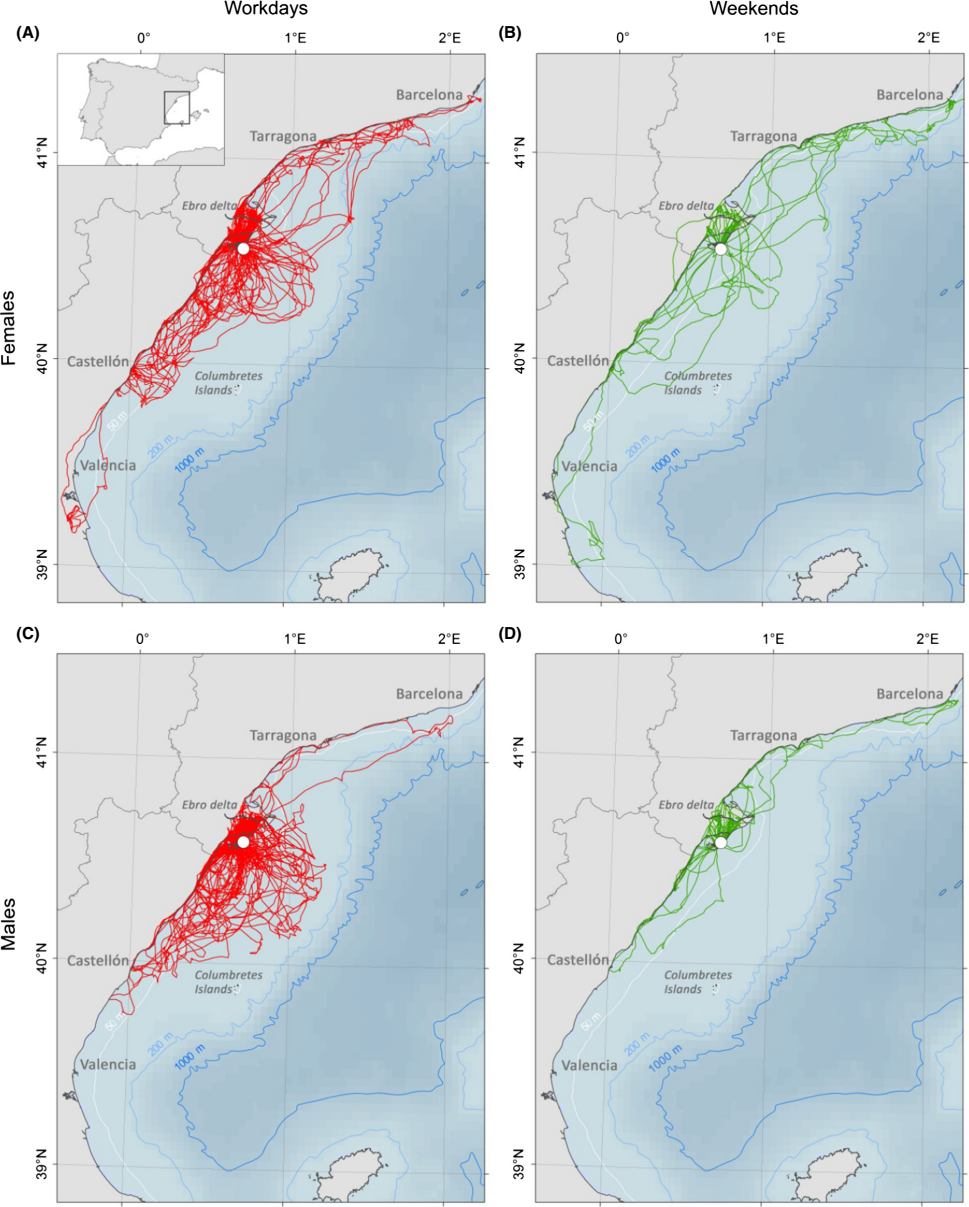
Foraging tracks of tagged Audouin's gulls from the Ebro Delta (Spain) by sex and fishing working cycles: (A) Females on workdays, (B) Females at weekends, (C) Males on workdays, and (D) Males at weekends.

Tagging provided 9 ± 5 (median ± IQR) complete days of suitable data from the individuals (max: 15 days; min: 1 day). Some devices were partially or totally unusable because of seal failure. The original sample data (*n* = 89,800 locations) were filtered to exclude incomplete days, resulting in 81,720 locations. Data from individuals remaining in the nest or colony were also excluded, so finally only the locations of foraging trips were used (*n* = 35, 154 locations). Foraging trips are defined as all locations from the moment the specimen leaves the colony until it returns, depicted in Figure1 (BirdLife International 2004).

Although four different types of habitats were identified for the Audouin's gull at the Ebro Delta (rice fields, sea, fishing harbors, and inland), in this study only rice field and marine habitats were considered because they accounted for more than 85% of locations (Navarro et al. 2010; García-Tarrasón et al. 2013).

To estimate individual foraging behavior, we considered: (1) *habitat use* as the proportion of rice field GPS locations over the sum of marine plus rice field positions each day; (2) *distance covered each day* (hereafter “daily distance,” in km) as the total cumulative linear distance covered in flights between all locations each complete day with available data; (3) *maximum distance from colony each day* (hereafter “maximum daily distance,” in km) as the linear distance between the furthest point of the trip and the nest for each complete day; (4) *trip length* (in km) as the total cumulative linear distance between all locations in each foraging trip; and (5) *trip duration* as the time lapse (in hours) between departure and return to the colony.

“Fishing activity” (workday or weekend) was assigned to every trip/day in order to evaluate a possible fishing effect. If a foraging trip lasted more than 1 day, including a workday and weekend, the assignment was made depending on where the individual spent most of this trip.

### Diet and stable isotope analysis

The heparinized vials were kept at +4°C and centrifuged (about 200 g for 10 min) within 6 h of collection. The supernatant plasma was pipetted off and stored in 250-*μ*L aliquots at −80°C. Plasma samples were lyophilized, manually homogenized and 0.3 mg were placed into tin capsules for stable carbon and nitrogen isotope ratio determination. Isotopic analyses were carried out at the Scientific and Technical Services of the Universitat de Barcelona (Spain) by means of a Thermo-Finnigan Flash 1112 elemental analyzer (CE Elantech, Lakewood, NJ) coupled to a Delta-C isotope ratio mass spectrometer via a CONFLOIII interface (Thermo Finnigan MAT, Bremen, Germany), with IAEA standards being applied every 12 samples to calibrate the system. Stable isotope ratios were expressed in the standard *δ* notation relative to Vienna Pee Dee Belemnite (*δ*^13^C) and atmospheric N_2_ (*δ*^15^N). Standard replicates indicated analytical measurement errors of ±0.1‰ and ±0.2‰ for *δ*^13^C and *δ*^15^N, respectively.

**Figure 2:**
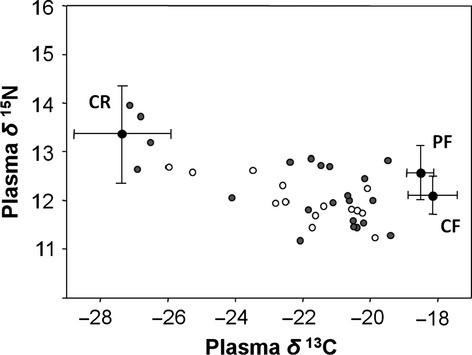
Scatter plot of the dispersion of *δ*^13^C/*δ*^15^N plasma values of Audouin's gulls at recapture. Males are shown as gray dots, females as empty dots. The mean and standard deviation of American crayfish (CR), Perciform fish (PF), and Clupeiform fish (CF) are also shown as potential prey. Isotopic data for the potential prey retrieved from García-Tarrasón et al. (2013).

For Audouin's gull prey, we used published reference values (mean ± SD) from the Ebro Delta following García-Tarrasón et al. (2013). Figure2 shows a graphical representation of individual Audouin's gulls and their potential prey. Lipids and uric acid concentrations in plasma can deplete *δ*^13^C values. For that reason, plasma *δ*^13^C values were corrected in Figure2, including +0.5‰ to compensate for the lack of lipid extraction in this tissue due to small quantities of plasma (Cherel et al. 2005). Potential prey was represented by adding an isotopic discrimination factor of +2.82‰ for *δ*^15^N and –0.08‰ for *δ*^13^C, following Caut et al. 2009.

Is worth to note that we did not consider to analyze red blood cells (RBC) isotopic ratios because it integrates a longer time frame (Votier et al. 2010) and we did not find it so much informative for this work. Audouin's gull is a migratory species wintering in Africa, so the isotopic analysis of the RBC at the capture event would probably integrated the diet of the wintering/migration period. In the case of the recapture event, the RBC probably overlapped too much with the plasma values.

### Condition of adults

Body mass at the recapture, body mass variation (capture and recapture weight difference), and a scaled mass index using the skull length as linear body measurement (following Peig and Green 2009) were used as condition indices.

### Statistical analysis

#### Habitat use and foraging behavior

Foraging behavior, on a trip or day basis, was modeled through generalized linear mixed models (GLMMs) including the individual as a random factor to account for dependence between observations made in the same bird. Sex and fishing activity (workday or weekend) were considered as fixed factors.

A logit link was used to model habitat use (proportion of GPS locations in a rice field per day), and an identity link was used to model continuous variables (daily distance, maximum daily distance, trip length, and trip duration) (Zuur et al. 2007). A hierarchical backward model selection, starting with a full model including both fixed factors and its interaction, was used. If the interaction factor was significant, a separate analysis was carried out for each sex. Distributional assumptions about data have been checked through graphical residual analysis. Foraging behavior variables showed clear skewed distributions that were normalized applying a logarithmic transformation.

#### SIA-GPS data relationship and consistence of diet strategy

The relationship between GPS data for habitat use (% of marine and % of rice field locations) and isotopic signatures of plasma at recapture (*δ*^13^C and *δ*^15^N) was tested using Pearson's correlation coefficient to assess the reliability of isotopes as indicators of foraging habitat.

We estimated consistency of individual diet strategy by comparing the plasma signatures between capture and recapture (ranging from 9 to 18 days). As blood plasma typically has a half-life of around 2–5 days, the signatures can provide dietary information in two nonoverlapping time periods (Podlesak et al. 2005). Isotopic signatures were also modeled using a GLMM with an identity link, including sex, capture event (capture vs. recapture), and their interaction as potential explaining factors. As capture and recapture isotopic values represent repeated measures for each individual, the repeatability of the individual feeding strategy was also estimated by calculating the intraclass correlation coefficient (ICC) and its associated uncertainty (Carrasco and Jover 2003; Votier et al. 2010). Individual consistence of foraging behavior (trip length and trip duration) and habitat use (rice field locations) was also evaluated using an ICC by sex and fishing activities (Stauss et al. 2012).

#### Individual condition and relationship with foraging behavior

A similar GLMM approach was used to investigate changes in body mass, which included the individual as a random factor, sex, and capture event as fixed factors, and the time span (in days) between capture and recapture as a covariate.

As body condition summarizes the costs (foraging effort) and rewards (food obtained) of the foraging strategy, we assessed the relationship between foraging effort (log of trip distance) and habitat use (using a non-GPS derived measure: the plasma *δ*^13^C at recapture) through a GLMM, including sex, fishing activity as well as the individual random factor.

The relationship between habitat use and different condition indices (body mass at recapture and the scaled mass index) was assessed performing a generalized linear model (GLM) including the sex as a fixed factor, and the habitat use (% of rice field locations) and time span between capture and recapture as covariates. In the case of body mass at recapture, the body mass at the initial capture was included as a covariate. The interaction of sex and habitat was also included.

All statistical analyses were performed using SPSS 21.0 (IBM Corporation, Armonk, NY).

## Results

### Habitat use and foraging behavior

When modeling the probability of rice field locations, the best model included only the fishing activity (*F*_1,31_ = 7.04, *P* = 0.012), indicating that the use of rice fields was higher at weekends than on workdays. There were no significant differences between sexes. Descriptive statistics (mean ± SD) are shown in Table1. The parameter estimate and 95% CI of the estimated effect are shown in the Figure S1.

**Table 1:** Descriptive statistics for the % of habitat use (marine or rice field) of Audouin's gulls by sex and fishing activities. Values are shown as mean ± standard deviation.

Sex	Fishing activity	% marine habitat	% rice field
Males	Workday	61.3 ± 39.3	38.7 ± 39.3
	Weekend	42.0 ± 40.6	58.0 ± 40.6
Females	Workday	64.5 ± 36.8	35.6 ± 36.8
	Weekend	58.5 ± 43.3	41.5 ± 43.3

In relation to foraging behavior (daily distance, maximum daily distance, trip length, and trip duration), all the selected final models (except the maximum distance) included the interaction between sex and fishing activity (all *P* < 0.036). This interaction was the result of significantly longer foraging trips in space and time undertaken by the females at weekends compared to males (Fig.1). In the case of the maximum daily distance, females foraged significantly further than males (*F*_1,1_ = 4.49, *P* = 0.043). Descriptive statistics (mean ± SD) are shown in Table2. The parameter estimate and 95% CI of the estimated effects are shown in the Figures S2–S5.

**Table 2:** Descriptive statistics for some foraging behavior parameters in the Audouin's gull. Values are shown as mean ± standard deviation. Maximum values are shown in brackets.

Sex	Fishing activity	Trip length (km)	Trip duration (h)	Daily distance (km/day)	Maximum distance (km)
Males	Workday	83.3 ± 71.0 [402.0]	7.2 ± 6.1 [39.6]	111.0 ± 61.7 [364.6]	35.8 ± 27.0 [145.2]
	Weekend	90.0 ± 89.2 [434.6]	9.6 ± 7.5 [32.9]	76.0 ± 51.6 [213.4]	33.2 ± 30.4 [149.8]
Females	Workday	94.3 ± 92.7 [533.5]	8.6 ± 8.5 [51.4]	126.2 ± 64.6 [282.9]	56.2 ± 43.3 [189.2]
	Weekend	160.7 ± 200.9 [771.4]	19.2 ± 20.2 [77.3]	130.5 ± 73.8 [259.8]	73.0 ± 56.7 [189.7]

*Trip length* was calculated as the total cumulative linear distance between all locations along the foraging trip. *Trip duration* was calculated as the time lapse between departure and return to the colony. *Daily distance* stands for the total cumulative linear distance covered flying between all locations each day with available data. *Maximum distance* was calculated as the linear distance between the furthest point of the trip and the nest for each day.

### SIA-GPS relationship data and consistence of diet strategy

GPS locations and plasma stable isotope values showed a full range of foraging strategies for this species, from rice field to marine specialists (Fig.2). Additionally, both GPS and plasma SIA data at recapture were highly correlated. The relationship of the % of rice field locations was significantly negative with the *δ*^13^C (*r* = −0.78, *P* < 0.001) and positive with the *δ*^15^N (*r* = 0.52, *P* = 0.002), and vice versa for the marine habitat.

**Table 3:** Descriptive statistics for the plasma stable isotopes (*δ*^13^C and *δ*^15^N) of Audouin's gull by sex and capture event. Values are shown as mean ± standard deviation.

	Sex	Mean ± SD
*δ*^13^C capture	Male	−22.69 ± 1.88
	Female	−23.14 ± 2.01
*δ*^13^C recapture	Male	−22.55 ± 2.53
	Female	−22.53 ± 1.89
*δ*^15^N capture	Male	12.41 ± 0.54
	Female	12.28 ± 0.58
*δ*^15^N recapture	Male	12.28 ± 0.77
	Female	12.00 ± 0.44

**Figure 3:**
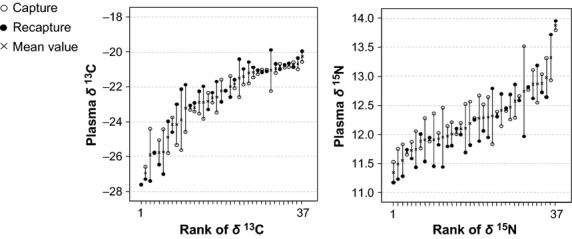
Paired plots showing plasma *δ*^13^C and *δ*^15^N between capture events. Each bar represents capture and recapture values observed for each individual. Individuals are ranked according to their mean value.

No significant gender differences were found for *δ*^13^C and *δ*^15^N between capture and recapture (Table3). Significant differences in nitrogen were found between both events (*F*_1,36_ = 5.43, *P* = 0.03). Moreover, *δ*^13^C and *δ*^15^N values showed a great repeatability between capture and recapture (*δ*^13^C: ICC = 0.78, 95% CI = 0.61–0.88; *δ*^15^N: ICC = 0.57, 95% CI = 0.31–0.76). Figure3 depicts a graphical approach to the isotopic variance components.

In the case of trip distance and trip duration, males on both workdays and weekends, and females on workdays showed a very low repeatability (trip distance ICC range; 0.12–0.22; trip duration ICC range: 0.00–0.15). However, females showed a great repeatability in their foraging behavior at weekends (trip distance: ICC = 0.97, 95% CI = 0.91–0.99; trip duration: ICC = 0.88, 95% CI = 0.62–0.97).

### Individual condition and relationship with foraging behavior

The distance covered on foraging trips was strongly correlated with habitat use. The best model included only the *δ*^13^C (*F*_1,28_ = 17.71, *P* < 0.001), indicating that a higher consumption of rice field resources was related to shorter foraging trips.

Differences in body mass were only found between sexes (*F*_1,36_ = 45.85, *P* < 0.001), but no significant differences were found between the two capture events. Despite the smaller flying effort required to move to neighboring rice field habitats, the final GLM for the body mass at recapture and the scaled mass index did not reveal any relationship with habitat use based on GPS data (all *P* ≥ 0.34).

## Discussion

Several studies have addressed the population ecology and behavior of the Audouin's gull as a model seabird (Oro and Ruxton 2001; Fernández-Chacón et al. 2013), although spatial distribution patterns have not been studied in much detail. Thanks to new techniques/devices, our results represent the most accurate approach to spatial patterns in foraging behavior in the Audouin's gull reported so far. Our contribution, together with previous knowledge, provides useful insights into the dependence of the Audouin's gull on fishing activities and the potential effect of a discard ban on the habitat use and scavenging ecology of this endangered species.

### Sex-specific foraging behavior in response to fisheries

Positional data indicate that female Audouin's gulls tended to perform longer foraging trips than males in both time and distance. This has already been suggested by Mañosa et al. (2004), despite the methodological and sample size constraints faced in their work. Studies on other seabird species have similarly shown that females usually perform longer foraging trips than males (e.g., González-Solís et al. 2000; Xavier and Croxall 2005). Spatial segregation by gender is widespread in seabirds, where the smaller sex (females in the case of the Audouin's gull) usually undertakes longer trips (reviewed in Wearmouth and Sims 2008). It has been suggested that size matters probably because the smaller and lighter sex has a higher foraging and flight efficiency (Shaffer et al. 2001). Foraging segregation has also been attributed to a reduced intraspecific competition (Catry et al. 2005). However, the sexual dimorphism hypothesis is not always supported (e.g., Lewis et al. 2002; Stauss et al. 2012). In fact, our data indicate that gender differences, that is, females traveling significantly longer distances and spending more time outside the colony, were only apparent at weekends, when there were no major fishing activities and therefore no discards.

### Individual specialists in a flexible population

The high correlation between GPS data and stable isotopes (especially carbon, which discriminates well between the use of marine and freshwater resources) indicates that the combination of both kinds of data provides a good description of the trophic ecology and show that habitat use results into effective tissue assimilation of isotopes. However, in contrast with the foraging trips, no significant interaction between sex and fishing activities was found in habitat use, possibly due to a high degree of individual variability in habitat use. Although both males and females increased their use of rice field resources in the absence of trawling activity, at the weekends males were observed to exploit this habitat more than females. A greater use of rice field prey in the absence of fishing activities has been reported before (Oro et al. 1996b), but not this sex-biased habitat selection (based on both foraging behavior and habitat use). However, no gender differences in plasma SIA were found, in contrast with Navarro et al. 2010. This means that on a larger timescale, traced by the stable isotopes, both males and females forage in the same habitats and consume equivalent prey during the incubation period.

Although both SIA and GPS data corroborate that Audouin's gulls exploit different habitats in the Ebro Delta, this apparent trophic plasticity hides a high level of individual specialization. Individuals ranged from being exclusively marine to rice field specialists, with all intermediate variations represented. Although a small decrease in the plasma nitrogen signatures was found between capture and recapture (ca. 0.2‰), both isotopes showed a great repeatability between capture events. The greater variation among rather than within individuals suggests a high consistency in habitat selection. Individual foraging specialization seems to be common in seabirds (e.g., Votier et al. 2004; Patrick et al. 2014) due to intraspecific aggregation and competition (Araújo et al. 2007), as well as the predictability of prey patches (Weimerskirch et al. 2007). In contrast, we found low repeatability in the foraging trips (trip length and duration), with the exception of females at weekends, when a great individual consistency in foraging trips was observed. Our results suggest that the gulls have far more foraging options at sea during workdays, when trawlers are operating and large amounts of discards are available. Then at the weekends, females continue showing a clear preference for the marine environment, given the high repeatability of long foraging trips. This is consistent with the hypothesis that females need to target specific resources after laying, and need to know where and when to find these resources in situations of food shortage.

### Does habitat use influence individual condition?

Foraging trip descriptors suggest that flying effort is highly related to the habitat where individuals forage. Energy spent foraging in rice fields is significantly lower than at sea, where gulls have to fly longer distances, following the fishing vessels or fishing by themselves. However, despite the different costs, no significant relationship between habitat use and condition indices was detected. Tentatively, this lack of correlation may indicate a possible trade-off between foraging effort and food quality: the higher energy cost of feeding at sea is offset by the superior nutritional quality of fish compared to American crayfish, and vice versa. However, other factors, such as lack of statistical power if this relationship is too weak, cannot be ruled out in this study.

Why then do females make a greater use of marine habitats than males in the absence of fishing activities? Although usually overlooked, female nutritional requirements after laying could play an important role in sex-specific foraging behavior (see, e.g., Lewis et al. 2002; Xavier and Croxall 2005). It is known that egg formation is a demanding period for avian females in general (Nager 2006) and Audouin's gull in particular (Ruiz et al. 2000), both in terms of energy costs and nutrient requirements. In this regard, some studies have shown that the need to restore calcium levels after laying could be an important explanatory factor related to sexual foraging segregation (reviewed in Wearmouth and Sims 2008). Indeed, calcium levels in Audouin's Gull females have been reported as a limiting factor that constrains egg synthesis (Ramirez et al. 2013). In this context, fish soft-bones represent a greater source of absorbed calcium than crustaceans (Hansen et al. 1998). Moreover, recent work has shown that antioxidants are limiting factors for breeding Audouin's Gull females and marine diets are important in providing eggs with a greater antioxidant capacity than rice field resources (García-Tarrasón et al. 2014).

## Conclusion

Although our results agree with previous studies showing that American crayfish act as a buffer for Audouin's gulls in absence of trawling activities (Oro et al. 1996b), the new data presented here on foraging behavior and habitat use reveal that crayfish are being differentially targeted by the sexes. Females perform longer at-sea foraging trips in situations of food shortage, probably because they need to meet specific nutritional requirements (energy content, micronutrients) associated with egg laying. Taking into account that Audouin's gulls are more likely to approach longline vessels when trawlers are not operating (Arcos and Oro 2002), and that this species is the second most affected seabird by longline bycatch in the Mediterranean (Laneri et al. 2010), a ban on discards could lead to an important sex-biased bycatch mortality (Báez et al. 2014). Additionally, individual cognitive abilities and specialization, such as learning how human activities modify the availability and predictability of food resources, may play a very important role in determining the foraging behavior of individuals (Cama et al. 2012; Oro et al. 2013). As these individual specializations may take several years to learn (Bicknell et al. 2013), any new policy needs to be implemented gradually to facilitate the adaptation of threatened species like the Audouin's gull to a discard ban scenario.

## Supporting Information

**Figure S1.** (A) Parameter estimate ± standard error for the logistic GLMM to fit the proportion of rice field positions over the total feeding positions. Weekends were used as a reference level in the analysis. (B) 95% CI of the estimated proportion of rice field positions derived from the model. **Figure S2.** (A) Parameter estimate ± standard error for the selected final GLMM in the log transformed distance covered per day (daily distance, in Km). Weekends and females were used as a reference levels in the analysis. (B) 95% CI of the estimated daily distance (in Km) derived from the model. **Figure S3.** (A) Parameter estimate ± standard error for the selected final GLMM to fit the log transformed maximum daily distance from the colony (in Km). Females were used as a reference level in the analysis. (B) 95% CI of the estimated maximum daily distance from the colony (in Km) derived from the model. **Figure S4.** (A) Parameter estimate ± standard error for the selected final GLMM in the log transformed trip length (in Km). Weekends and females were used as a reference levels in the analysis. (B) 95% CI of the estimated trip distance (in Km) derived from the model (in logarithmic scale). **Figure S5.** (A) Parameter estimate ± standard error for the selected final GLMM in the log transformed trip duration (in hours). Weekends and females were used as a reference levels in the analysis. (B) 95% CI of the estimated trip duration (in hours) derived from the model (in logarithmic scale).
