# Supplementary material for: Sex-specific foraging behavior in response to fishing activities in a threatened seabird
Source: Ecol Evol. 2015 May 22;5(12):2348–58. doi: 10.1002/ece3.1492 (PMC4475368; doi:10.1002/ece3.1492)
Supplement: Supplementary file 1 — Figure S1. (A) Parameter estimate ± standard error for the logistic GLMM to fit the proportion of rice field positions over the total feeding positions. Weekends were used as a reference level in the analysis. (B) 95% CI of the estimated proportion of rice field positions derived from the model. Figure S2. (A) Parameter estimate ± standard error for the selected final GLMM in the log transformed distance covered per day (daily distance, in Km). Weekends and females were used as a reference levels in the analysis. (B) 95% CI of the estimated daily distance (in Km) derived from the model. Figure S3. (A) Parameter estimate ± standard error for the selected final GLMM to fit the log transformed maximum daily distance from the colony (in Km). Females were used as a reference level in the analysis. (B) 95% CI of the estimated maximum daily distance from the colony (in Km) derived from the model. Figure S4. (A) Parameter estimate ± standard error for the selected final GLMM in the log transformed trip length (in Km). Weekends and females were used as a reference levels in the analysis. (B) 95% CI of the estimated trip distance (in Km) derived from the model (in logarithmic scale). Figure S5. (A) Parameter estimate ± standard error for the selected final GLMM in the log transformed trip duration (in hours). Weekends and females were used as a reference levels in the analysis. (B) 95% CI of the estimated trip duration (in hours) derived from the model (in logarithmic scale). [file ece30005-2348-sd1.docx]

**Supplementary Material**

**Sex-specific foraging behaviour in response to fishing activities in the threatened Audouin’s gull**

Manuel García-Tarrasón^1,*^, Juan Bécares^2^, Santiago Bateman^1,2^, José Manuel Arcos^2^, Lluís Jover^3,4^ and Carolina Sanpera^1,4^

^1^Departament de Biologia Animal (Vertebrats), Facultat de Biologia, Universitat de Barcelona, 08028 Barcelona, Spain

^2^ SEO/BirdLife, 08026 Barcelona, Spain

^3^ Departament de Salut Pública, Facultat de Medicina, Universitat de Barcelona, 08036 Barcelona, Spain

^4^Institut de Recerca de la Biodiversitat (IRBio), Universitat de Barcelona, 08028 Barcelona, Spain

*Corresponding author at: Departament de Biologia Animal (Vertebrats), Facultat de Biologia, Universitat de Barcelona, Avinguda Diagonal 643, 08028 Barcelona, Spain. Tel.: +34 93 4021041; fax: +34 93 4035740.

E-mail address: m.garcia@ub.edu (M. García-Tarrasón).

**Figure S1.** (A) Parameter estimate ± standard error for the logistic GLMM to fit the proportion of rice field positions over the total feeding positions. Weekends were used as a reference level in the analysis. (B) 95% CI of the estimated proportion of rice field positions derived from the model.

**B**

**A**


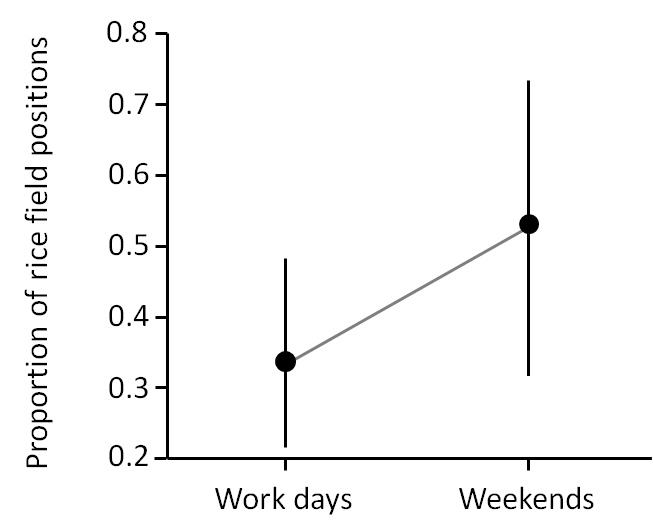


|  | Logit Proportion  of rice field positions |
| --- | --- |
| Intercept | 0.127 ± 0.432 |
| Work days | -0.798 ± 0.301 |
| Weekends | Ref. |
| Model variances |  |
| Individual | 3.036 ± 0.869 |
| Residual | 29.697 ± 2.436 |

**Figure S2.** (A) Parameter estimate ± standard error for the selected final GLMM in the log transformed distance covered per day (daily distance, in Km). Weekends and females were used as a reference levels in the analysis. (B) 95% CI of the estimated daily distance (in Km) derived from the model.

**B**

**A**


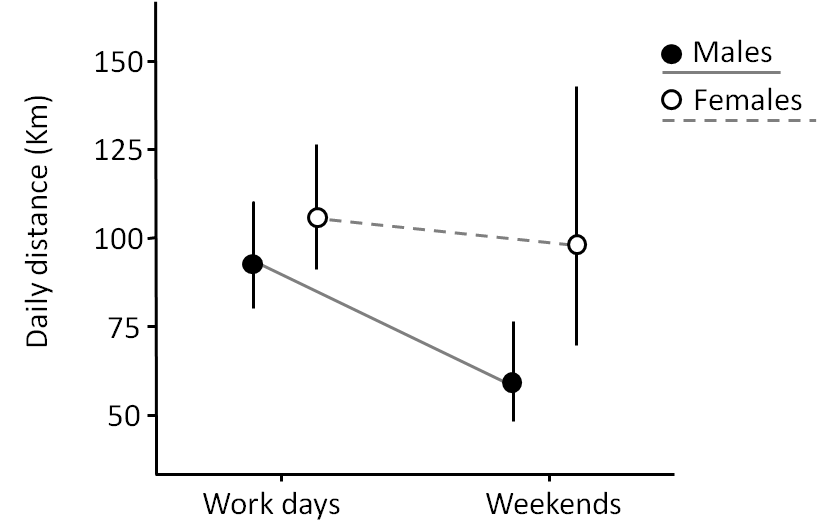


|  | Log Daily Distance |
| --- | --- |
| Intercept | 1.999 ± 0.078 |
| Work days | 0.032 ± 0.060 |
| Weekends | Ref |
| Males | -0.217 ± 0.093 |
| Females | Ref. |
| Work days * Males | 0.160 ± 0.074 |
| Model variances |  |
| Individual | 0.015 ± 0.006 |
| Residual | 0.052 ± 0.005 |

**Figure S3.** (A) Parameter estimate ± standard error for the selected final GLMM to fit the log transformed maximum daily distance from the colony (in Km). Females were used as a reference level in the analysis. (B) 95% CI of the estimated maximum daily distance from the colony (in Km) derived from the model.

**B**

**A**


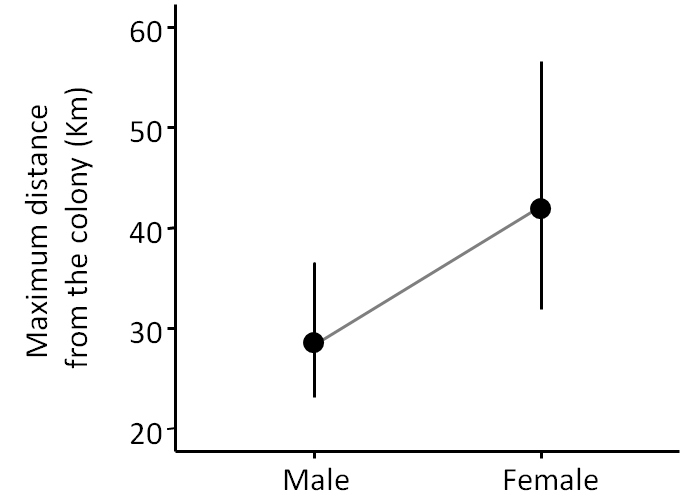


|  | Log Maximum Daily Distance  from the Colony |
| --- | --- |
| Intercept | 1.628 ± 0.013 |
| Males | -0.164 ± 0.077 |
| Females | Ref. |
| Model variances |  |
| Individual | 0.041 ± 0.869 |
| Residual | 0.061 ± 0.005 |

**Figure S4.** (A) Parameter estimate ± standard error for the selected final GLMM in the log transformed trip length (in Km). Weekends and females were used as a reference levels in the analysis. (B) 95% CI of the estimated trip distance (in Km) derived from the model (in logarithmic scale).

**A**

**B**


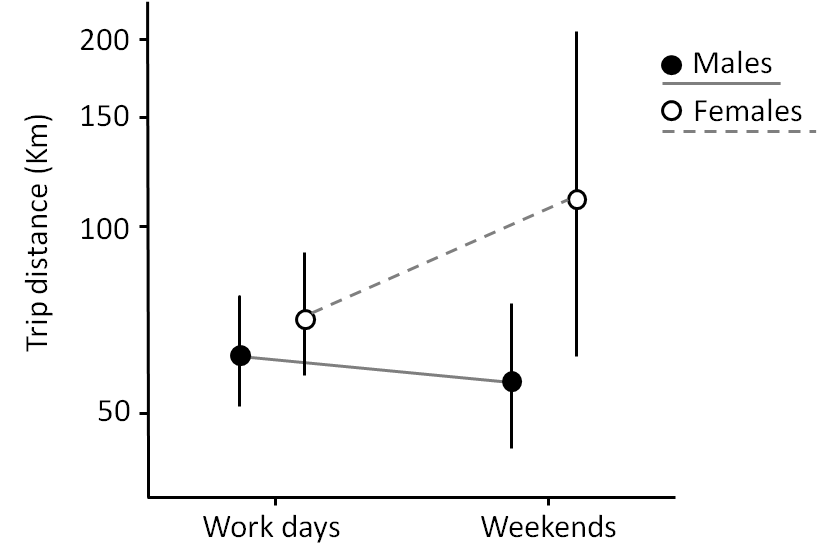


|  | Log Trip Length |
| --- | --- |
| Intercept | 2.052 ± 0.129 |
| Work days | -0.193 ± 0.093 |
| Weekends | Ref |
| Males | -0.294 ± 0.142 |
| Females | Ref. |
| Work days * Males | 0.234 ± 0.101 |
| Model variances |  |
| Individual | 0.029 ± 0.011 |
| Residual | 0.119 ± 0.010 |

**Figure S5.** (A) Parameter estimate ± standard error for the selected final GLMM in the log transformed trip duration (in hours). Weekends and females were used as a reference levels in the analysis. (B) 95% CI of the estimated trip duration (in hours) derived from the model (in logarithmic scale).

**B**

**A**


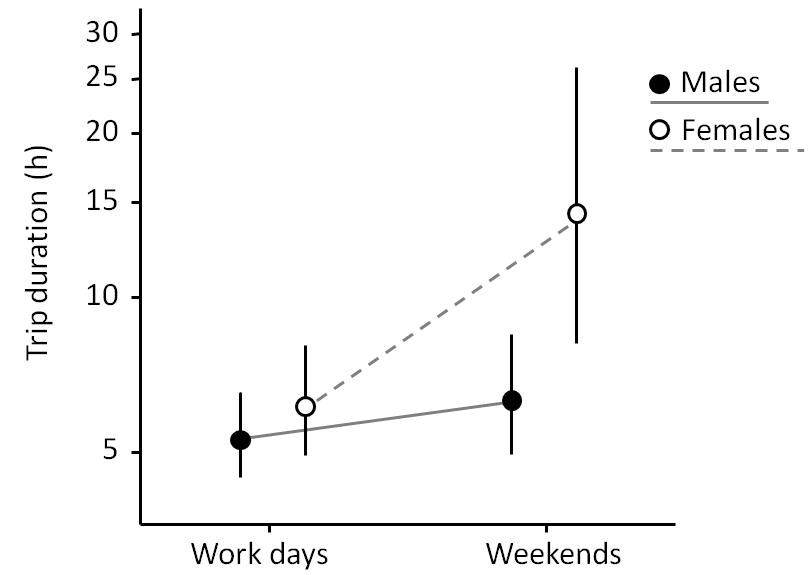


|  | Log Trip Duration |
| --- | --- |
| Intercept | 1.165 ± 0.126 |
| Work days | -0.364 ± 0.103 |
| Weekends | Ref |
| Males | -0.353 ± 0.140 |
| Females | Ref. |
| Work days * Males | 0.284 ± 0.117 |
| Model variances |  |
| Individual | 0.025 ± 0.010 |
| Residual | 0.139 ± 0.011 |

**BIOSKETCH**

**Manuel García-Tarrasón** is interested in the interdisciplinary combination of intrinsic biogeochemical markers (such as trace elements and stable isotope analysis), physiological markers and tracking devices in order to deepen in the knowledge of a wide range of ecological processes in seabirds such as feeding ecology, pollutant accumulation, oxidative stress studies, spatial distribution and habitat use. This study is part of his PhD Thesis titled *Trophic Ecology, Habitat Use and Ecophysiology of Audouin’s gull (*Larus audouinii*) in the Ebro Delta*.

Author contribution: M.G., J.B., J.M.A., L.J. and C.S. conceived the ideas; M.G., J.B., S.B. and J.M.A. collected the data; M.G., J.B. and L.J. analysed the data; and M.G., L.J. and C.S. led the writing.
